# Resilience in keeping the balance between demand and capacity in the COVID-19 pandemic, a case study at a Swedish middle-sized hospital

**DOI:** 10.1186/s12913-023-09182-4

**Published:** 2023-02-28

**Authors:** Ritva Gisela Rosenbäck, Ann Svensson

**Affiliations:** 1 Department of Engineering Science, University West, Trollhättan, Sweden; 2 School of Business, Economics and IT, University West, Trollhättan, Sweden

**Keywords:** Pandemic, COVID-19, Resilience, Healthcare, Surge capacity, Balance

## Abstract

**Background:**

In pandemics, it is critical to find a balance between healthcare demand, and capacity, taking into consideration the demands of the patients affected by the pandemic, as well as other patients (in elective or emergency care). The purpose of this paper is to suggest conceptual models for the capacity requirements at the emergency department, the inpatient care, and intensive care unit as well as a model for building staff capacity in pandemics.

**Methods:**

This paper is based on a qualitative single case study at a middle-sized hospital in Sweden. The primary data are collected from 27 interviewees and inductively analyzed.

**Results:**

The interviewees described a large difference between the immediate catastrophe scenario described in the emergency plan (which they had trained for), and the reality during the COVID-19 pandemic. The pandemic had a much slower onset and lasted longer compared to, for example, an accident, and the healthcare demand fluctuated with the societal infection. The emergency department and inpatient care could create surge capacity by reducing elective care. Lower inflow of other emergency patients also helped to create surge capacity. The number of intensive care beds increased by 350% at the case hospital. At the same time, the capacity of the employees decreased due to infection, exhaustion, and fear. The study contributes to knowledge of conceptional models and key factors affecting the balance between demand and capacity.

**Conclusion:**

The framework suggests conceptual models for balancing surge capacity during a pandemic Health care practitioners need to provide assumptions of the key factors to find the balance between the demand and capacity corresponding to the reality and maintain the delivery of high-quality healthcare services.

## Background

### Introduction

The COVID-19 pandemic placed extraordinary constraints on healthcare services due to increased demand, especially at intensive care units (ICU), combined with limited capacity [1]. Healthcare organizations needed to rapidly build surge capacity, which is defined as the ability of healthcare services to expand their operations to safely treat an abnormally large influx of patients in response to a disaster [2–4]. The increased demand in the pandemic directly impacted the limited resources that healthcare services build on, such as staff and stuff (equipment, materials, and drugs) as well as structure and system [2].

During the pandemic, healthcare organizations all over the world struggled to provide care to patients infected by the COVID-19 virus, while at the same time providing normal healthcare to other patients during a longer period [5]. To create surge capacity for COVID-19 patients, hospitals needed to use capacity intended for other patients. To effectively utilize the total capacity at hospitals, it is important to create balance. Hick et al. [2] claims there are potentially large gaps in the ability of healthcare systems to find sufficient capacity to cope with large-scale infectious disease outbreaks.

Pandemics tend to come in waves associated with fluctuations in demand, which require staff and other resources to be available continuously and the surge capacity to be adjusted often. Thus, the concept of resilience, i.e., following a shifting demand, could be better applied in pandemics [6–10].

Most research on surge capacity in disasters concerns the management of short-term disasters that develop fast [3, 4, 11, 12]. In short-term disasters, the capacity needs to rise quickly to a high level but lasts just a few hours or days. In pandemics, on the other hand, the capacity needs to last much longer. Balancing staff capacity and demand for healthcare services is the most challenging part of enduring long-term crises for healthcare organizations [3].

In the very beginning of the COVID-19 outbreak in Europe, on the 5th of March 2020, a letter was published by three professors from Milan in northern Italy [13]. The letter warned that up to 10% of the infected citizens would need respirators, which would create a huge surge demand for ICU beds. They sent a message to the world: “We wish to convey a strong message: Get ready!“.

### Aim

The novel paper focuses on the balance between the demand for healthcare and the capacity of staff to provide healthcare during a longer period of time (in this case, the COVID-19 pandemic). It is important to learn from the COVID-19 pandemic and document the new knowledge to boost resilience and make us better prepared for future pandemics [9, 14–16].

Suggestions regarding surge capacity in pandemics are before the COVID-19 pandemic purely theoretical, and more evidence-based research is needed to guide the development of effective surge capacity [2, 17, 18]. This paper focuses on how to practically keep the balance between demand and capacity of staff in order to maintain healthcare services during pandemics, such as COVID-19. The aim of the paper is to propose a conceptual framework to understand and manage the balance between the healthcare demand and the capacity of staff at the emergency department (ED), inpatient care, and ICU at hospitals. To be able to achieve the balance in a pandemic, it is necessary to understand the factors needed to calculate the staff capacity requirements from patient demand, and the factors to build the capable capacity of staff,. This understanding is needed to help the strategic organizational leadership obtain more accurate information and create resilience [9, 16, 19].

The research question is: Which key factors are important to consider when balancing between capacity and demand in pandemics?

### Literature review

The demand needs to be balanced with the available capacity of various resources [20]. It is well known that the demand for healthcare is fluctuating over time, which causes significant challenges for the healthcare sector [21–23]. The demand expressed as number of patients in need of healthcare needs to be translated to capacity requirements, which could be for example number of care days at inpatient care or at the ICU, to be able to dimension the right capacity [20]. A specific characteristic of the service sector, such as healthcare organizations, is that the demand needs to equal the available capacity, as capacity of healthcare services cannot be stored. Both demand and capacity are time dependent [24]. It is also important to consider where and when to deliver the resources, and how much resources to deliver to ensure a certain service level [25].

Various researchers have proposed frameworks for capacity planning in healthcare systems. Vissers et al. [26] proposes a hierarchical framework for planning and control at all levels. However, this framework seems to be based on a quite stable number of available resources and stable demand for healthcare services during a long time period without disruptive changes. Bonnett et al. [4] proposes a conceptual framework for surge capacity, which addresses the progression of surge responses after a disaster. In later research, the concept of healthcare resilience is used for describing the management of a disaster or pandemic [7] for example during the Ebola epidemic [6].

Building surge capacity is vital when the medical and healthcare needs of the patients exceed the existing resources and the overall ability of the healthcare system to manage a disaster [2–4]. Thus, the healthcare system must shift the way it delivers healthcare, from day-to-day operations to expanding the capacity to manage a surge of patients that goes beyond a normal situation [4]. The need for surge capacity varies depending on the type of disaster. There could be situations where a shift occurs, from the daily goal of triage, to providing care to the sickest patients first, to “doing the greatest good for the greatest number of patients” [3, p. 1159]. Strategic use of resources must benefit the population, rather than individual patients [12]. To build surge capacity and maintain it over days or years, healthcare services must be prepared and make proactive decisions. Healthcare services also need to maximize the use of available resources, which are often limited, to save lives [17].

Surge capacity depends on four essential components: (1) staff (with appropriate knowledge and experience), (2) stuff (equipment, materials, and drugs), (3) structure (physical structure), and (4) system (management infrastructure, strategies for communication, etc.), according to Faccini et al. [27] and Hick et al. [2]. Managing the surge capacity implies effectively matching the available resources to an increasing or changing demand for the resources. Building surge capacity can require difficult decisions about how to allocate scarce resources, especially staff [12]. Shortage of staff can make it difficult to maintain usual standards of healthcare services, especially regarding isolation capacity and critical care [2].

Therrien et al. [7] merges the concepts of surge capacity and resilience, and develops the concept of surge capacity with resilience factors in the healthcare sector. Healthcare resilience includes both proactive factors, related to improving the preparedness, and reactive factors, which are used when needed. Sundararaman et al. [8] also uses resilience to describe preconditions for healthcare during the COVID-19 pandemic. Anderson et al. [9] and Sanford et al. [16] use resilience as a concept to evaluate the imagine of the work, how the work is done in reality, and how successful the work is, in order to improve the adaptiveness and quality. Resilience is also needed at national and international levels [10, 28].

Deschepper et al. [29] suggests a tool for planning how many beds are needed at different wards. The tool is built on a Poisson model of admitted patients, offers a planning horizon of about ten days, and can be used at the operational level. Elkhuizen et al. [30] describes how to foresee the amount of staff needed at different wards based on bed utilization and nurse-patient ratios, both in the short and long term. This paper describes how to translate epidemiological prognoses of infection spread to needed hospital capacity, in order to build the right surge capacity in pandemics with rather short planning horizons.

## Methods

### Research approach

This paper builds on a single case study. An inductive approach, and a process of building theory from case study research, inspired by Eisenhardt [31] and Carroll and Swatman [32], was used. The research method consists of several steps, including literature review, definition of the research question, case selection, interviews, analysis, interpretation of the results, reflections on the results, and comparison with existing literature. A case study produces context-dependent knowledge and experiences from the recent COVID-19 pandemic [33], and can be used to understand how the case achieved balance between capacity and demand. The research is influenced by the interpretive perspective, described by Klein and Myers [34], as it focuses on understanding context-dependent complexity and the process of human sense making in balancing demand and staff capacity in pandemics.

The literature review identified gaps in the existing research about surge capacity and resilience and provided understanding of current knowledge and theories within this area. The data collection and data analysis were performed in iterations that overlapped in time until closure with saturated data was reached. The findings were then compared with the literature to challenge and find support for the knowledge building and framework creation. The initial research question was refined during the iterations, as the data were compared with the emergent themes and literature. The inductive research approach is applicable since “advances in knowledge that are too strongly rooted in what we already know delimit what we can know” [35, p.16]. Research on surge capacity in pandemics can add new knowledge to the more researched field of surge capacity for disasters.

Cases which are likely to give the most information and extend the emergent theory should be selected, according to Eisenhardt [31]. Flyvbjerg [33] claims that extreme cases often reveal more context-dependent information. Therefore, a case hospital in Sweden with an early and severe outbreak of COVID-19, was chosen. The case is a middle-sized hospital with about 1,300 employees, located in a region with a high infection rate of COVID-19 at the time of the study. The hospital collaborated with other hospitals in the region in terms of dividing the work and sharing the burden.

### Data collection

The data were collected by semi-structured interviews [36, 37], to be able “to obtain both retrospective and real-time accounts by those people experiencing the phenomenon of theoretical interest” [35, p.19]. All interviews were recorded and transcribed verbatim. This approach was used to interpret the social phenomena and show what is socially constructed by the managers and key employees at the case hospital [38]. The data collection focused on acquiring a deep insight and understanding of the surge capacity planning during the COVID-19 pandemic. While the COVID-19 pandemic affected the whole case hospital, three departments were most affected: the ED, the inpatient care unit, and the ICU. The study includes interviews with 27 interviewees in total, including members of the hospital management group including the CEO, CMO (chief medical officer), managers of operations, human resources, communication, education, and care. Outside the management group the unit managers of the ED, inpatient care, and ICU were also interviewed, as well as service managers handling logistics and maintenance, who contributed to building surge capacity. Two interviews (with unit managers) were conducted in groups of two (i.e., two persons were interviewed at the same time). All other interviewees were interviewed alone. A total of 25 interviews were conducted through a video conference tool, each of which lasted for about an hour. The same two researchers conducted all the interviews (although neither were not always present): an “involved researcher” and an “observing researcher”. The “involved researcher”, who is knowledgeable in the research context, moderated the dialogues, and the observing researcher mainly listened, ensured that the interview guide was followed, and added a few questions. The interview sessions started in March 2021, one year after the start of the pandemic and during the third wave. All 25 interviews were completed three months later. The interviews contained questions regarding the size of pandemic, emergency plan, building and balancing surge capacity, management, and communication. Ethical review was not conducted since the interviews did not contain any sensitive personal questions. Informed written consent to participate in the study was obtained from all participants, who had received information about the study beforehand via a letter.

### Data analysis

The interviews were transcribed and inductively coded in order to be able to build theory objectively, through close adherence to the data [39]. Many codes were associated with the first-order findings adhering to the informants, as expected [35]. The researchers read, reread, and discussed the transcripts to gain a deep understanding of the data. In the search for similarities and differences among the codes, they were aggregated into groups that were assigned less specific second-order codes. Examples of first-order codes for the theme balance are seen in Table 1. Other first-order codes were processed in the same way and grouped into other themes, as for example “demand”, “capacity”, “hospital management”, “leadership”, and “staff”. These themes should be able to describe and explain the observed phenomenon in a more theoretical way [35], and link the findings to the aim of the research [32]. The data codes and themes were compared to existing literature in the process of exploring the suggested factors in the conceptual framework.

**Table 1:** Example of codes within the theme “Balance”

Example quotes from the interviews	Codes	Theme
“We did not manage the higher healthcare level all by ourselves due to the hospital’s fundamental mission – we needed help from the University hospital.”	Capacity shortage	Balance
“There were a lot of relocations of employees, staff members to other units, we needed to do it to strengthen the needing units, but that decreased the elective care a lot and for quite a long time.”	Decrease of elective care	
“There is a special group for planning the number of surgery teams – but then we need to ensure that inpatient resources exist. I think it has worked well.”	Increase of elective care	
“The first wave was worse than the second wave.”	Excess capacity during the first wave	

As the data analysis was done partly in parallel to the data collection, the researchers were able to adjust the interviews along the way, by, for example putting more emphasis on specific themes that emerged as important. Both researchers analyzed the data iteratively in a circular process of developing codes and themes from the data. The researchers continued to discuss the interpretations of the data, codes, and themes while reporting the research findings. The involved researcher provided significant feedback to the management group at the hospital and the results were discussed and validated by them, as recommended by Walsham [40].

## Results

The interviewees described a large difference between the immediate catastrophe scenario described in the emergency plan, which they had trained for, and the reality during the COVID-19 pandemic. The pandemic had a much slower onset and lasted longer compared to, for example, an accident, and the demand for healthcare fluctuated with the societal infection. As one of the managers described it:

It’s not a point, it’s a line.

The letter published in Italy [13] marked a starting point in a chain of actions. A fast decision was made, about two weeks before the first patients were admitted to the hospital, to increase the case hospital’s intensive care capacity. As one interviewee from the ICU said:

Lucky circumstances, a brave CEO, and a bit of luck with timing, with the right person at the right place so we could get momentum in this and get started.

### Demand and capacity requirements

The first wave of COVID-19 in Sweden started in early March 2020. At the same time, the flow of all other emergency patients decreased, due to fear of infection. The CEO of the hospital pointed out:

Other emergency patients didn’t come, they disappeared from the Earth.

As the hospital stopped all elective care in March 2020, making the total capacity requirement of inpatient care low. Soon after, in April 2020, the patient inflow peaked and almost two thirds of the patients were hospitalized into inpatient care or ICU. As one interviewee said:

Almost exactly a year ago – in early April, there around Easter last year – then it was a very difficult situation.

The societal infection of COVID-19 decreased during the summer, but in late October 2020, the second wave emerged. The third wave started right after, in February 2021, and ended in late spring, leaving only a few patients with COVID-19 at the hospital in the beginning of July. The interviewees found the pandemic surprisingly demanding, considering the slow start, the multiple waves, the fast decrease of demand at the end of the waves, and the long duration.

The capacity requirements at the ED were slightly higher than normal during the pandemic. Less need to treat non-COVID emergency patients was compensated by greater need to treat COVID-19 patients. The flow of patients from the ED to inpatient care was much faster than normal since the inpatient care unit maintained an overcapacity and the ED reduced the diagnose processes. The average care time decreased from several hours to as low as about fifteen minutes during the first wave. Later, the average care time increased slightly due to faster COVID tests, thus conducted at the ED. The flow of other emergency patients increased during later waves to about 80% of the normal level.

The capacity requirements at the ICU were huge in all three waves, due to a high inflow of COVID-19 patients in need of intensive care and since the average care times were longer than normal. The chief physician said:

It’s not the usual ICU treatment times we talk about, they can stay for fourteen days.

The reduction in number of other emergency patients and elective surgery only had a negligible effect on the ICU capacity requirements.

### Surge capacity building

Since the COVID-19 pandemic affected the entire world, it was difficult to source staff capacity from other hospitals (which can sometimes be done otherwise). The case hospital had seven means of increasing staff capacity; having staff work overtime, reducing time for vacation and studies, sourcing staff from other units where the demand for healthcare was lower (voluntarily or involuntarily), sourcing staff from private healthcare providers, renting nurses, and employing new staff and volunteers without any healthcare knowledge. During the first wave, staff were obliged to relocate to the ED, inpatient care, and the ICU. Fewer staff relocated during later waves because the obligation was replaced by a voluntary possibility to do so, and many staff hesitated because they were not sure they had enough knowledge. Therefore, the units that treated the COVID-19 patients received less help from other units of the hospital in the second and third waves.

Reduced healthcare demands of non-COVID patients and short average care times meant that the surge capacity at the ED did not need to be considerably enlarged. Relocated doctors and nurses helped to triage infectious as well as noninfectious patients. The need for surge capacity at inpatient care increased as a result of stricter security routines, but due to reduced demand, it was enough to increase the number of staff by 15% (mostly rental nurses and relocated outpatient clinic nurses).

The on-call lines of medicine physicians, treating medicine patients in inpatient care and at the ED during nights, had to be increased from two to three. The internal medicine department’s physicians worked a lot of overtime even though they received valuable help from other specialist physicians. The manager of medicine specialists said:

We all helped each other, and the cooperation was high, on top.

The maximum staffed capacity at the ICU was 18 beds with respirators, which was 350% more than the hospital’s normal capacity of four beds. The number of staff increased from 180 to 310 in three weeks, which was very challenging for the organization. The ICU nurses received help from relocated nurses, most of whom came from the hospital’s surgery department. New employees without healthcare education also joined the staff. These so called “ICU assistants” received special education and training about intensive care and hygiene procedures to be able to help the care nurses with trivial tasks. Specialist physicians from other specialties took care of more stable patients.

Absent staff do not contribute to the capacity. Therefore, it was important to consider the staff’s wellbeing. Psychologically, there was an obvious risk of exhaustion due to high work pressure associated with treating severely ill patients and lack of knowledge. As an interviewee said:

”Great need for crisis support, not for relatives and patients and so on – the patients too, of course, but our staff, above all, need crisis support because they are not feeling well in this.”

Physically, there was a risk of being infected with COVID-19, but other physical problems also arose, such as weight gain, diabetes, and wear-out injuries from using protection equipment. The management and staff of the case hospital were occupied with preventing the infection from spreading between patients and between patients and staff, while the risk of infection spread among the staff was in the beginning neglected. The coffee rooms were crowded, the management team met in a small “crisis management room” and protective care equipment education was conducted in large mixed groups in small conference rooms. Many interviewees commented:

How could we do that?

The hospital suffered several infection outbreaks: for example, one among the members of the management team at Easter 2020 and another at the surgery department at the end of October. After the outbreak in October, the hospital began conducting so called hygiene rounds in coffee and conference rooms, and restrictions on number of people allowed in rooms were set. In 2021, after it had become obligatory the wear masks and the hospital staff had been vaccinated, the sick leave among the employees returned to a normal level.

### Balancing capacity and demand

To continuously establish a balance between the demand and capacity, the hospital developed causality planning. During the first wave, the causality was set to increase stepwise up to 100% of the Italian scenario. At the same time, a causality planning group was mobilized that compiled daily hospital statistics and gathered information about the societal infection spread. The causality planning group increased the causalities stepwise from 50% up to 100% of the demand of the first wave, and used it as a base for making decisions to build surge capacity in the next couple of weeks.

The ED managed to balance capacity and demand throughout the waves, thanks to relatively fast outflow of patients to inpatient care or home, and the possibility to ask for help at the daily meetings. As a manager at ED said:

As long as the outflow of patients to the wards is working, we will manage. (…) We didn’t use much extra staff during wave two and three, because we knew that if we asked for more staff before the daily meeting, we would get it.

The inpatient care unit needed to be very flexible and keep the balance between COVID care and other care by changing focus. After wave one, the number of inpatient care beds per hospital to be used for COVID care was stated each week on an overall regional level, but as the inpatient unit manager said:

I haven’t really cared so much about what the region said about the amount of COVID beds we should have – for me it’s more important that we have places for the patients who come. Honestly.

During the infection peaks, the healthcare demand exceeded the capacity of the ICU. More than one patient per day was moved from the hospital to a nearby university hospital in order to try to achieve more balance. Most patients were moved to the university hospital in April 2020 (a total of 70, according to the hospital statistics). In the first wave, before the peak, the ICU management decided to overstaff to be better prepared for higher demand. Sometimes it even became too crowded, or as one ICU manager expressed it:

It was not possible to reach the bed of a very ill patient because of the amount of people in the room.

When the knowledge of capacity requirements increased, the number of redundant staff was reduced.

As the infection waves subsided, the ED first experienced lower demand, followed by inpatient care. Due to the long hospitalization of the patients at the ICU and the expansion over their normal capacity, it took about two weeks before the ICU also experienced a normal demand. The elective surgery could not restart before this, because their staff worked at the expanded ICU. The decision to start elective surgery was therefore owned by the ICU. As the chief physician at the ICU said:

It’s hard to decrease the beds, because it’s like a tail: when the number of COVOD-19 patients started to decrease, ICU still had a pressure.

Another ICU manager said:

For us, the pandemic is still in the different stages, but the chirurgic and orthopedic departments are just waiting for us to say ok.

On the other hand, the elective patients also needed to be handled to lower the risk that their conditions deteriorated, especially as the pandemic was prolonged. As the manager of the surgery department at the hospital said:

We sometimes think we have our values – ‘the patient first’ – it has almost become the COVID patient first – which I sometimes think one should think about. What have we put in the other scale? What will we see in the future? There will certainly be studies on which patients have suffered.

**Fig. 1:**
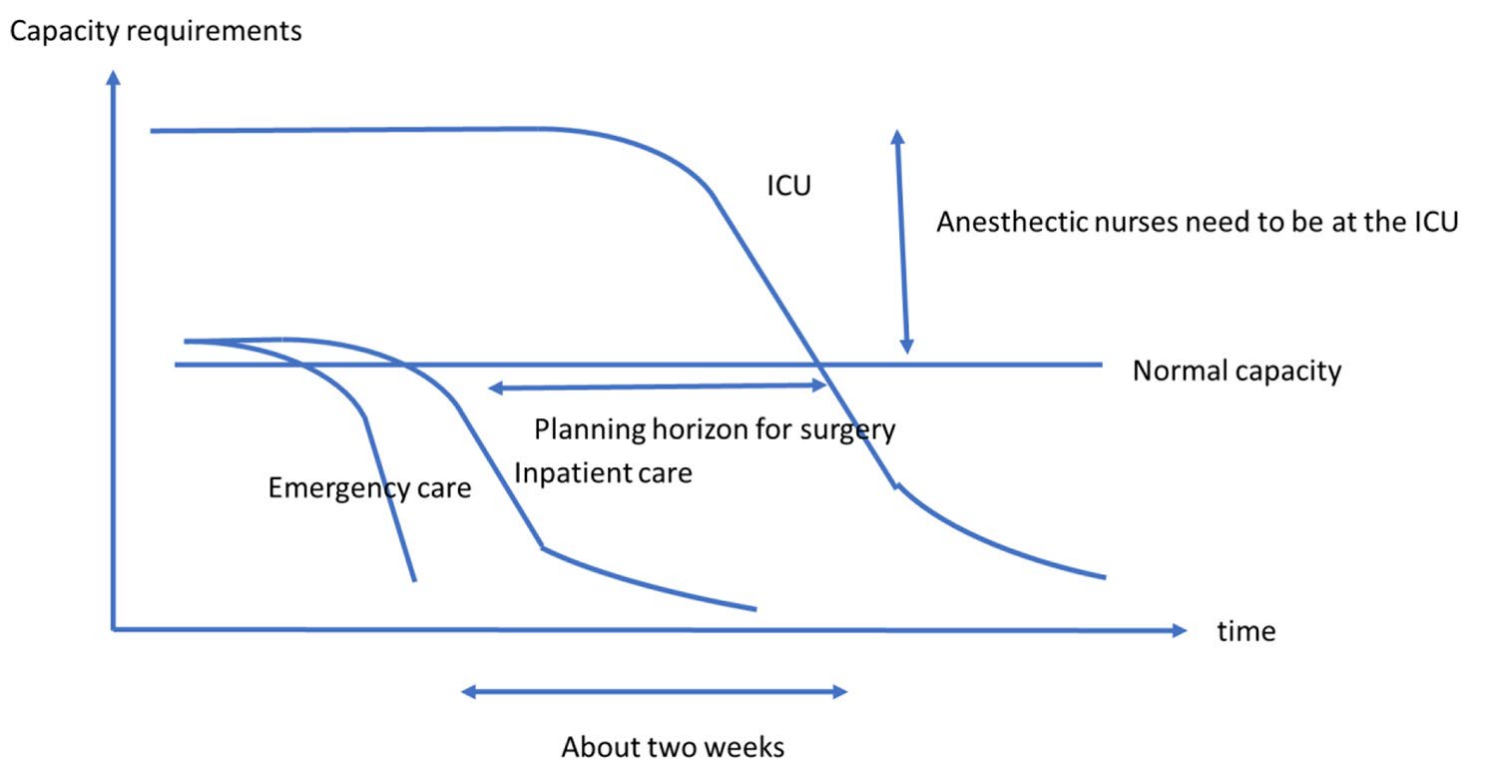
Schematic illustration of the need for capacity over time at the emergency department, inpatient care, and intensive care unit at the receding end of an infection wave

Figure 1 shows a schematic diagram built on the findings. The time scale of the diagram shows that the need for capacity decreased at the ED and inpatient care about two weeks before it decreased at the ICU. The planning was complicated by the fact that the surgery department needed a planning horizon of about two weeks to prepare the patients for surgery.

The daily planning, both at the strategic level and the tactical level, was the most important factor in balancing the loading of COVID-19 patients, other emergency patients, and elective patients at the case hospital and the other hospitals in the region.

## Discussion

In the beginning of a pandemic, there is little knowledge about how much and what type of staff capacity the patients will need. It is also uncertain how much capable capacity the healthcare system can provide to meet the demand. It is of major importance to achieve a resilient balance between capacity and demand of healthcare services to be able to save as many lives as possible, as Hick et al. [17] describes.

More knowledge will be gained in the following waves and the suggested conceptual models, outlined in previous sections, could help to make daily predictions of the capacity needed to balance the demand. The suggested conceptual models take into consideration factors to dimension the capacity requirements, expressed as days or hours of care, based on data of spread of infection (expressed as number of patients per time unit), following Little’s law [41]. The focus of capacity requirements are sensitive in healthcare organizations culture, but the practitioner’s prediction of how the average care time or the quota of patients needing intensive care are essential to determine the total required capacity [20]. To determine the resilient balance in production of healthcare services, the suggested models include a dimensioning of the capable capacity as shown in previous sections.

### The effect of the COVID-19 pandemic on demand and production of healthcare

In the beginning of the pandemic, the healthcare demands of non-COVID emergency patients decreased at the case hospital. During later waves, it increased to 80% of the normal level. The demand for elective care was managed by the hospital by, for example, decreasing the number of elective surgeries, visits, and treatments. Elective care also decreased naturally as a result of ill peoples reluctancy to visit the hospital. To what extent it is possible to reduce elective activities is of course a medical tradeoff between how dangerous an infection is compared to other sicknesses. Frail elderly who passed away as a result of infection led to a noticeably lower demand for both emergency and elective healthcare later in the COVID-19 pandemic.

To ensure that the demand can be managed during an unknown first wave, it seems advisable to build up excess capacity at the affected units. Balancing before the peak of the first wave includes being ready to execute the next step in the causality planning to maintain the safe overcapacity. It is important to analyze from the beginning how the pandemic is likely to affect the normal emergency healthcare and how a decrease in elective healthcare could contribute to the surge capacity.

### Conceptual model of capacity requirements

**Fig. 2:**
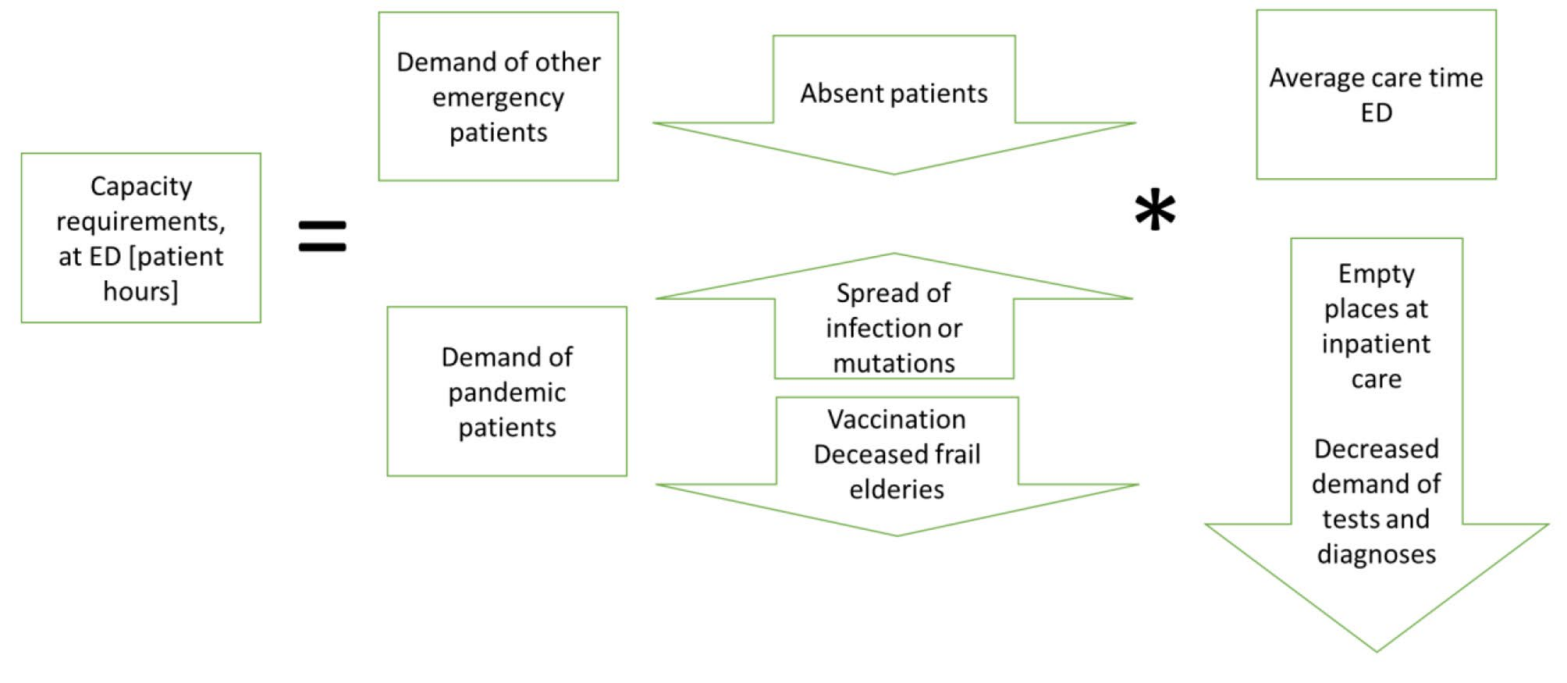
Conceptual model of capacity requirements at the ED.

**Fig. 3:**
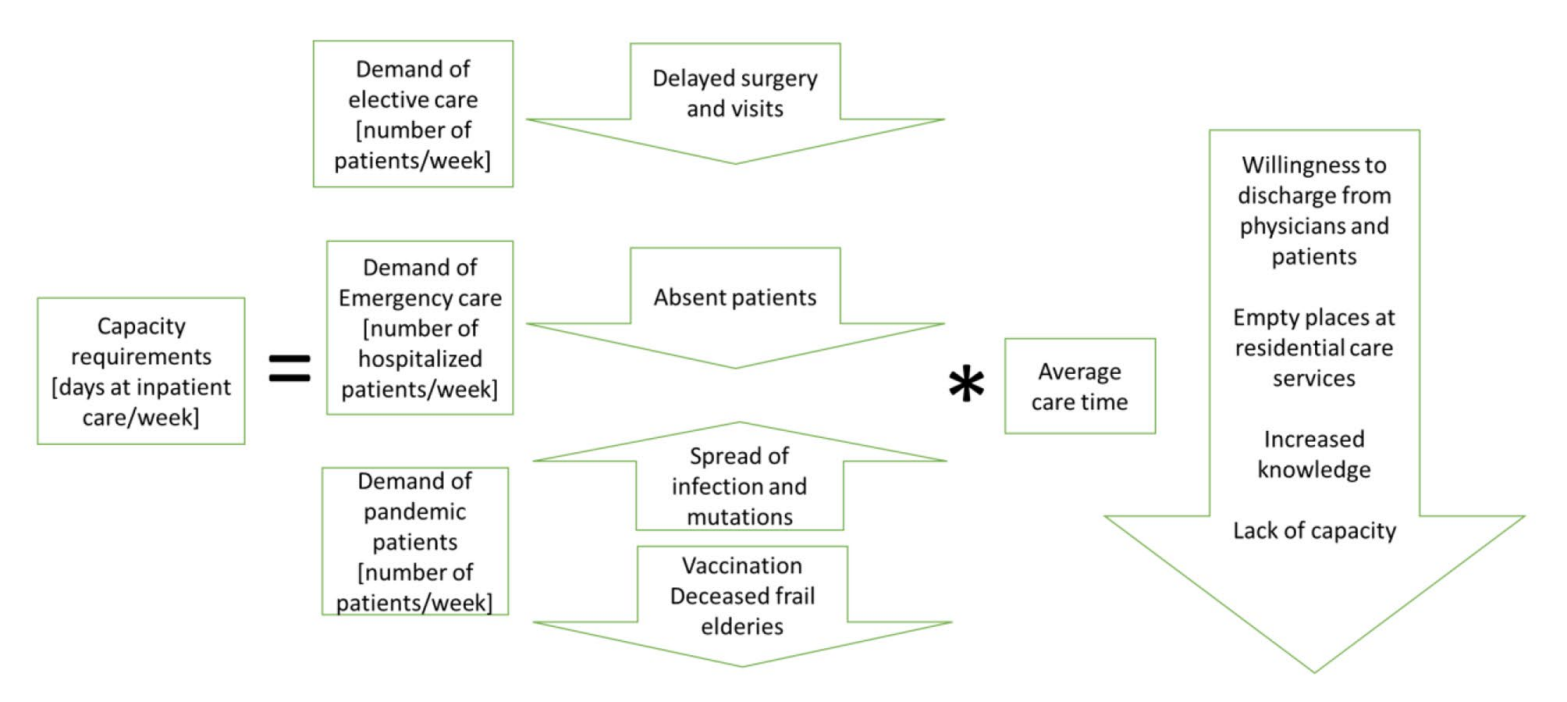
Conceptual model of capacity requirements at the inpatient care

The total capacity requirements at different care units are estimated from the demands of the pandemic patients, other emergency patients, and elective care patients (see Figs. 2 and 3). The factors are illustrated with a probable increase or decrease (arrow up or down). The demands of the pandemic patients follow the societal infection waves and may decrease after some time due to vaccination and immunity. The demands of other emergency patients decrease as a result of fear to visit the hospital and because people in risk groups pass away. The same goes for elective patients, but the level could also be changed and balanced by the management.

When dimensioning the capacity requirements at the ED the inflow of patients and the average care time needs to be considered (Fig. 2). Empty beds at the inpatient care and lower demand for tests and treatments before patients are hospitalized decreases the average care time at the ED.

Figure 3 describes the demand for inpatient care. The average care time of other patients at inpatient care could be reduced by discharging patients from the hospital as soon as possible. The average care time could also be shorter, caused by the physicians reluctance to overload the inpatient care. As elderly pass away as a result of infection, more free places will become available at short and long residential care services. Elderly who have recovered after treatment at the hospital can then go to residential care services after discharge. The average care time decreases as knowledge about treatments of the infection increases.

The ICU care demand could be dimensioned from the inpatient care demand by multiplying the number of COVID-19 patients at the inpatient care with the actual ICU hospitalization coefficient and the actual average care time at the ICU. The actual coefficients varies during pandemics due to mutations of the virus and increased knowledge about the infection.

### Conceptual model of building surge capacity

**Fig. 4:**
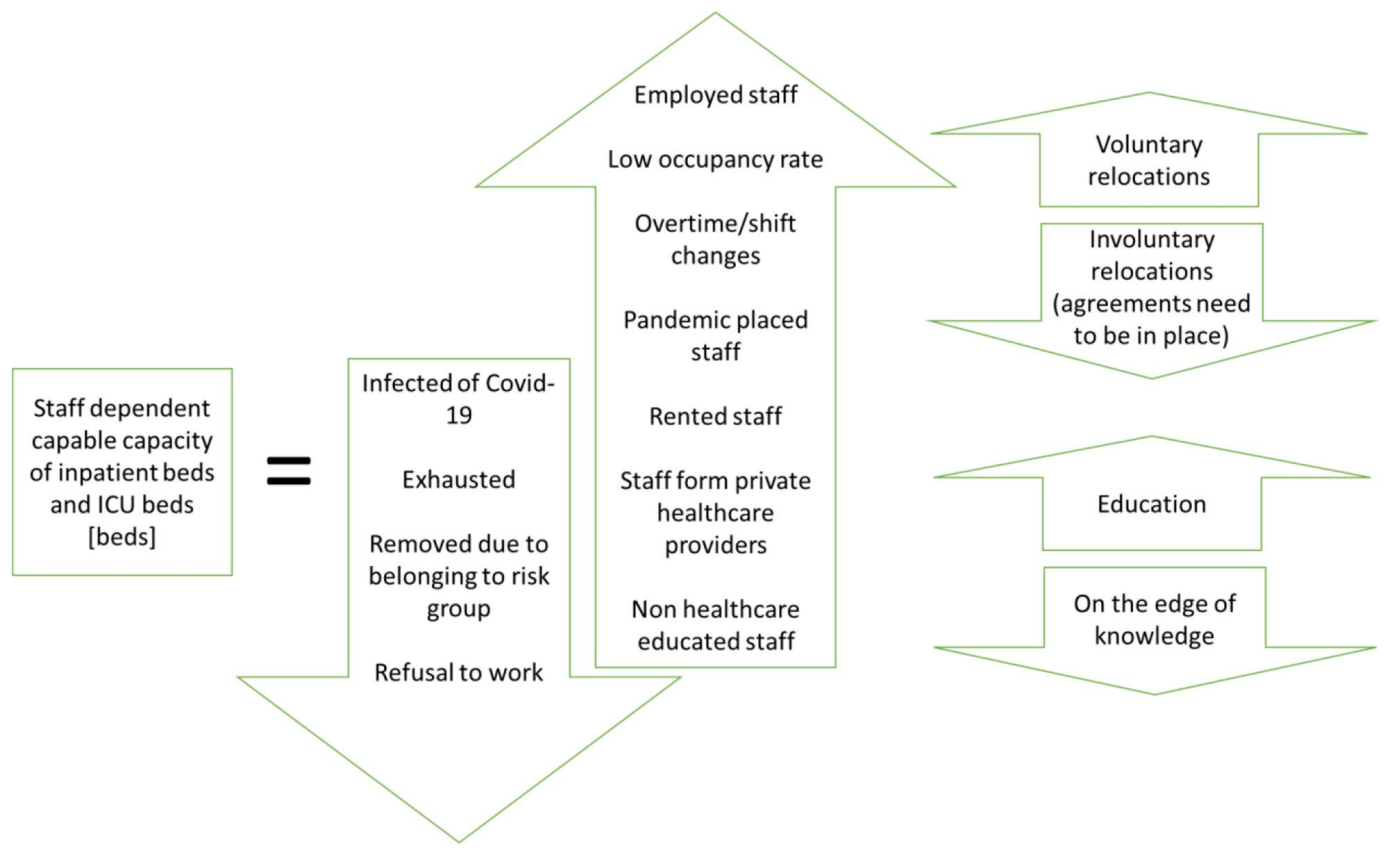
Conceptual model of capable capacity of inpatient and ICU beds

The long-term building of surge capacity to handle pandemic patients depends on how the critical healthcare units can be staffed. It is important to identify the critical units that need surge capacity, for example an inpatient care unit or the ICU, as well as the units with reduced demand. The units with reduced demand, for example elective treatments or visits, could provide capacity to the starving units. Figure 4 shows a model with factors for building capacity. As Therrien et al. [7] describes, the organization needs to be resilient in finding time (overtime, shift changes, etc.), spreading out the most knowledgeable and specialized staff over time and place, and sourcing as knowledgeable staff as possible from different healthcare providers (staff relocated using a voluntary or involuntary approach, rented staff, and staff from private healthcare providers). If the demand is very high, staff with lower healthcare knowledge can be used as assistants for trivial tasks, in order to use the most knowledgeable staff more effectively. This strategy situation is also described by Bonett et al. [4]. All relocated and newly employed staff need to be educated to do their tasks. It is important to carefully divide and thoroughly describe the tasks, to reduce the education time and use all staff as effectively as possible.

It is of major importance to consider the risk of losing staff capacity during a pandemic. The reduction of capacity could be caused by the infection itself, exhaustion, an ambition to protect staff risk group members, or refusal to work.

The building of surge capacity can be simplified if agreements that specify how staff relocations should be made and how hospital internal staff and private healthcare providers should be compensated are in place beforehand. If the relocations are discussed and decided before, the staff could be educated and trained, and thus be better prepared, which Hick et al. [12], Therrien et al. [7] and Sundararaman et al. [8] also discuss. Moreover, it is advisable to be well staffed to be better prepared; at least not understaffed at normal demand, as Hick et al. [2] also points out.

The models developed here consider the capacity of the units affected by the COVID-19 pandemic, namely the ED, the inpatient care, and the ICU. The next pandemic could of course require other type of surge capacity. How a future pandemic might affect other units is not included in the models presented here.

### Balance

**Fig. 5:**
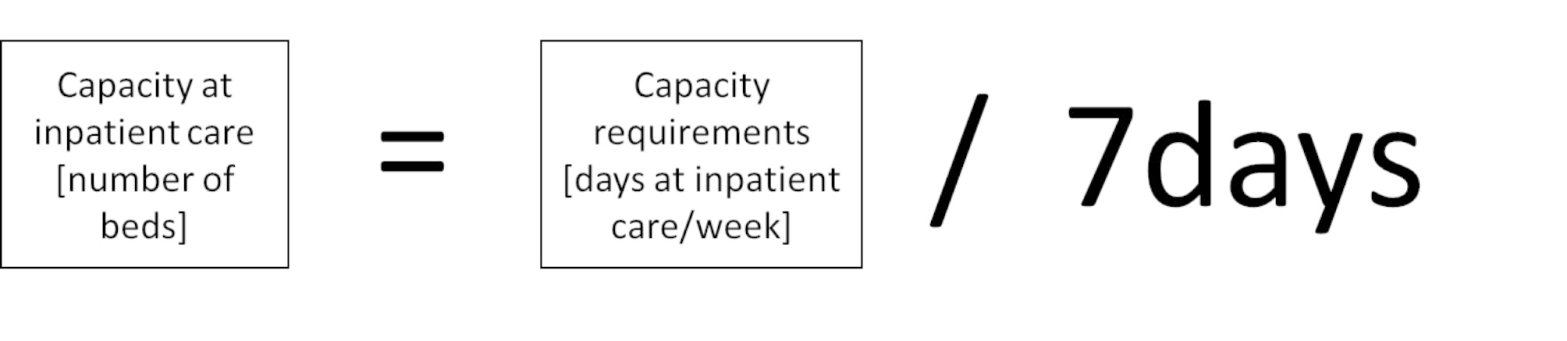
Calculation of the balance

The capacity requirements in the model (Figs. 2 and 3) shown as care days per week for different inpatient care or patient hours at the ED must be transferred to the same unit as the capacity. The model of balance for inpatient and ICU care thus needs to be divided by the days the unit is open per week (i.e., seven days), see Fig. 5. For the ED, the total hours of all patients at the unit need to be spread out during the day, and expressed as patient hours per day hour, and then compared to the average possible number of patient hours the staff could handle.

## Conclusion

The conceptual models suggested in this paper describe the key factors that are important to consider when balancing between capacity and demand in the healthcare production during pandemics. During a pandemic, the numerous of the factors need to be considered daily by healthcare practitioners to make the balance correspond to reality.

At the case hospital studied here, the ICU needed the most surge capacity in the COVID-19 pandemic. The inpatient care and the ED partly built the surge capacity they needed from the reduction of normal (non-COVID) emergency patients and elective care.

It is important to protect the staff from infection, both from the patients and from each other, in order to keep the capable capacity. The staff’s psychological health, which seems to be as important as their physical health, can be protected with adequate education, access to relevant information and knowledge, and support from management and therapists.

The conceptual models are developed from one single case study and could be improved by testing the models in a complementary case study. It would be interesting to conduct a quantitative survey at several hospitals in different regions and countries to be able to generalize the importance of the factors in the models. Some aspects are of special interest – for example the shorter planning horizon and the integration between different decision levels. It would also be interesting to investigate if the reduction in number of (non-COVID) emergency patients and delayed elective care have resulted in any patient injury. Last but not least, It would be interesting to return to the case hospital after one or two years to see what changes still remain.

## Data Availability

The datasets generated and/or analyzed in the current study are not publicly available since they contain data in the form of interviews in a survey. The interviews have been transcribed and anonymized during the process. The transcribes are available from the corresponding author upon reasonable request.

## Declarations

### Authors’ information

Ritva Rosenbäck is a Ph.D. student of Engineering system at University West, Sweden. Her research interests are production and operations logistics in healthcare organizations. She also serves as a managerial consultant within the area of strategic, tactic and operational management and planning in healthcare organizations in Sweden.

Ann Svensson is Professor of Informatics and Work-Integrated Learning at University West, Sweden. Her research interests are within knowledge management, learning and collaboration in digitalization, with particular focus on professional work within and across organizations. She currently studies healthcare organizations. She also serves in editorial board of journals and conferences, as well as the director of Health Academy West, Sweden.
